# Enhancing study recruitment through implementation of an opt-out, cold contact process with consideration for autonomy, beneficence and justice

**DOI:** 10.1017/cts.2023.21

**Published:** 2023-02-08

**Authors:** Tara Pittman, Leslie Bell, Stedman Jones, Kimberly Brown, Katie Kirchoff, Patrick Flume

**Affiliations:** 1 South Carolina Clinical & Translational Research (SCTR) Institute, Medical University of South Carolina, Charleston, SC, USA; 2 Biomedical Informatics Center (BMIC), South Carolina Clinical & Translational Research (SCTR) Institute, Medical University of South Carolina, Charleston, SC, USA; 3 Department of Medicine and Pediatrics, Medical University of South Carolina, Charleston, SC, USA

**Keywords:** Clinical trial recruitment, research recruitment, patient portal, electronic health record, cold contact, contact preference, cold calling, patient engagement, opt-out, recruitment strategy

## Abstract

The potential utilization of a cold-contact approach to research recruitment, where members of the research team are unknown to the patient, has grown with the expanded use of electronic health records (EHRs) and affiliated patient portals. Institutions that permit this strategy vary in their implementation and management of it but tend to lean towards more conservative approaches. This process paper describes the Medical University of South Carolina’s transition to an opt-out model of “cold-contact” recruitment (known as patient outreach recruitment or POR), wherein patients can be contacted so long as they do not express an unwillingness to receive such communication. The work highlights the benefits of this model by explaining how it, in many ways, supports and protects autonomy, beneficence, and justice for patients. The paper then describes the process of standing up the recruitment strategy, communicating the change to patients and the community, and documenting study team contact and patient research preference. Data supporting increased access to potentially eligible patients of greater diversity as well as initial researcher feedback on perceived success of POR is also shared. The paper ends with a discussion of next steps to enhance the POR process via more detailed data collection and reengagement with community stakeholders.

## Introduction

Electronic medical records (EMRs) typically include patient portals creating an additional opportunity for clinicians to engage directly with patients. Consequently, investigators have identified the potential for this type of patient engagement to enhance and expand recruitment strategies [1]. Even when investigators have no established relationship with potentially eligible patients, they could invite them to participate in research, a recruitment approach borrowed from commercial settings also known as cold contact [2]. Utilization of cold contact as a recruitment tactic varies amongst academic medical centers [3]. Some institutions use an opt-in approach to cold contact. This approach is more conservative in that it requires patients to actively designate their willingness to receive contact about research from unknown parties. A limitation of this strategy is that it typically results in fewer patients eligible for contact and may lead to potential selection bias [4–6]. Conversely, an opt-out approach implies that an institution defaults to permitting cold contact for all patients in the health system unless a patient expresses an unwillingness to receive such contact [7]. Although patient privacy and burden are valid concerns of an opt-out, cold contact approach to recruitment, a comprehensive dissemination of research opportunities is vital to achieving diversity in research. In fact, it can be argued that the principles outlined in the Belmont Report can be used as a framework for highlighting the strengths of an opt-out approach, as this approach addresses concerns about patient autonomy (e.g. offering patient choice and control), beneficence (e.g. reducing selection bias), and justice (e.g. reducing lack of generalizability) [3]. In addition, opt-out models, when implemented, are generally well received by both clinicians and patients and show promise for higher recruitment rates and more representative samples [8 ,9,^10^].

Historically, recruitment of patients for research purposes at the Medical University of South Carolina (MUSC) had been limited to the clinical care relationship; that is, investigators were only able to recruit their own patients to their clinical trials. Attempts were later made to capture a patient’s research contact preference during the clinical consent process at visit check-in. However, this felt like an inappropriate time for such a question and it was difficult to track the stated preferences in a way that could be stored and communicated across the enterprise. When MUSC increased its promotion and adoption of MyChart^™^ (Epic’s patient portal system), it enabled their Epic Research team to create a Research Preferences Questionnaire which was pushed to all MUSC MyChart^™^ users, asking whether they did or did not want to be contacted about potential research studies for which they might qualify; patients could also indicate they were not ready to make a decision at that time. Reminders to complete the questionnaire were pushed at various intervals, and patients were asked to re-set their preferences annually.

Through this mechanism, MUSC effectively became an opt-in institution with regard to cold contact recruitment. This resulted in a small number of patients being eligible for contact since only a small fraction of MUSC patients were using MyChart^™^ and an even smaller fraction of those patients answered the questionnaire. Consequently, the goals of increasing dissemination of research opportunities and enrolling more diverse and representative samples were not fully achieved. In response, MUSC’s institutional leadership gave the South Carolina Clinical and Translational Research (SCTR) Institute (an NIH-funded Clinical and Translational Science Award [CTSA] hub) approval to spearhead the transition to an opt-out approach for cold contact recruitment. MUSC’s cold contact recruitment process, referred to as patient outreach recruitment (POR), designed to increase reach and opportunity for patient participation in research, is presented below.

## Methods

A Research Preferences Manager was hired to spearhead and manage the development of the opt-out recruitment infrastructure. Key stakeholders of the POR process were identified, and a steering committee was developed to oversee the initiative and guide decisions made throughout the process. Steering committee members included representatives from the university compliance office, the Institutional Review Board (IRB), legal counsel, Biomedical Informatics (BMIC), Epic Research, and institutional research leadership.

The process included an initial review of existing practices among other academic medical centers as well as meeting with key advisors including MUSC leadership and the Community Engagement (CE) and Integrating Special Populations (ISP) cores of SCTR. The goal of these initial procedures was to get feedback on how to capture public opinion related to contact about research opportunities, establish trust with the community, and create and disseminate messaging about this new policy both internally and publicly. Two participant engagement groups (PEGs) [11], resembling focus groups and modeled after Vanderbilt Institute for Clinical and Translational Research’s Community Engagement Studios, were conducted to get feedback on the aforementioned points from two groups: patients and community members, and trusted leaders from across the state (e.g., physicians, church leaders, educators, and leaders of prominent community organizations).

The following section of this paper highlights the key findings of initial research and feedback on the establishment of an opt-out approach and the resulting messaging, institutional partnerships, and workflows related to process implementation. In addition, initial data related to patient identification and access, as well as researchers’ perceptions of the utility of POR for study enrollment are shared.

## Results

### PEG Feedback

PEG participants in both groups provided feedback on the following: who/what they looked to locally for trusted health information, a draft research website for the lay public (with information about research participation, research contact, and instructions for patients opting-out of research contact), and a draft of the required phone script to be read by research teams contacting patients using the POR strategy. The “trusted leader” PEG was also asked about resources they would need to bring information about research opportunities, participation, and contact to their specific communities. Both sets of PEG participants emphasized the importance of placing messages in a variety of communication channels, beyond just MUSC-initiated, patient-directed messaging; this would allow people throughout the state, including those not seen at MUSC, to be exposed to the new approach to research contact, helping establish trust and transparency, and sharing MUSC’s commitment to providing research opportunities for everyone. Thoughts and feedback provided during these PEGs were used to inform communication and messaging, opt-out documentation methods, and changes to MUSC’s recruitment policy.

### Communication and Messaging

Messaging was placed throughout the health system (on digital announcement boards in clinics and vertical banners in high-traffic campus areas) and pushed out through hospital newsletters, social media, and employee communication channels. Additionally, messaging was distributed through statewide media channels via commercials, local news interviews, newspaper ads, and science podcasts during the months prior to the POR launch.

### Opt-out Documentation

**Fig. 1.**
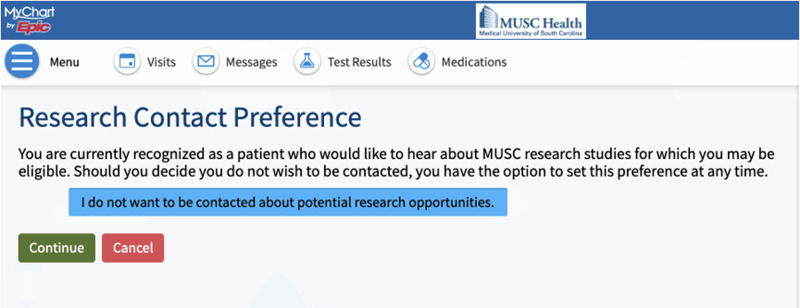
Research preferences questionnaire in MyChart^™^.

An opt-out version of the Research Preferences Questionnaire in MyChart^™^, shown in Fig 1., was created to allow patient portal users a mechanism to decline receiving cold contact about research opportunities. The form contains a single opt-out selection but also allows patients to deselect their opt-out status should they change their preference. Patients who had previously indicated that they did not want to be contacted (in the original opt-in Research Preferences Questionnaire) had that preference mapped to the opt-out selection in the new questionnaire.

The patient-facing research website was updated to contain information about all methods to opt-out. Given the limited patient engagement with MyChart^™^, it was deemed necessary that patients have additional methods of opting-out. Patients can contact the Research Preferences Manager at any time to update their contact preference; they can also opt-out of all future research contact via study personnel when they are contacted about a specific study. Unless a patient independently expresses a desire to opt-out of research contact, they are never prompted by study team members or institutional messaging to document a research preference or to indicate that they prefer not to opt-out of contact, as this would more closely resemble an opt-in strategy requiring each patient to give permission prior to being contacted about research.

### Cold contact as a Recruitment Strategy

To provide framework for cold contact, the IRB’s recruitment policy was updated, and the POR process was created, which includes steps to apply for, utilize, and document cold contact recruitment. Guidance documents to assist investigators with this process were posted to SCTR’s recruitment resource website. Any MUSC researcher who wants to directly contact patients (via phone call, email, letter, MyChart^™^, etc.) about research with whom they have no established clinical connection must follow the POR process. That being said, while POR is available to all MUSC investigators, it may not be a suitable strategy for every project. Therefore, the IRB determines the appropriateness of using POR for each unique protocol.

### Requesting Patient Contact Lists

**Fig. 2.**
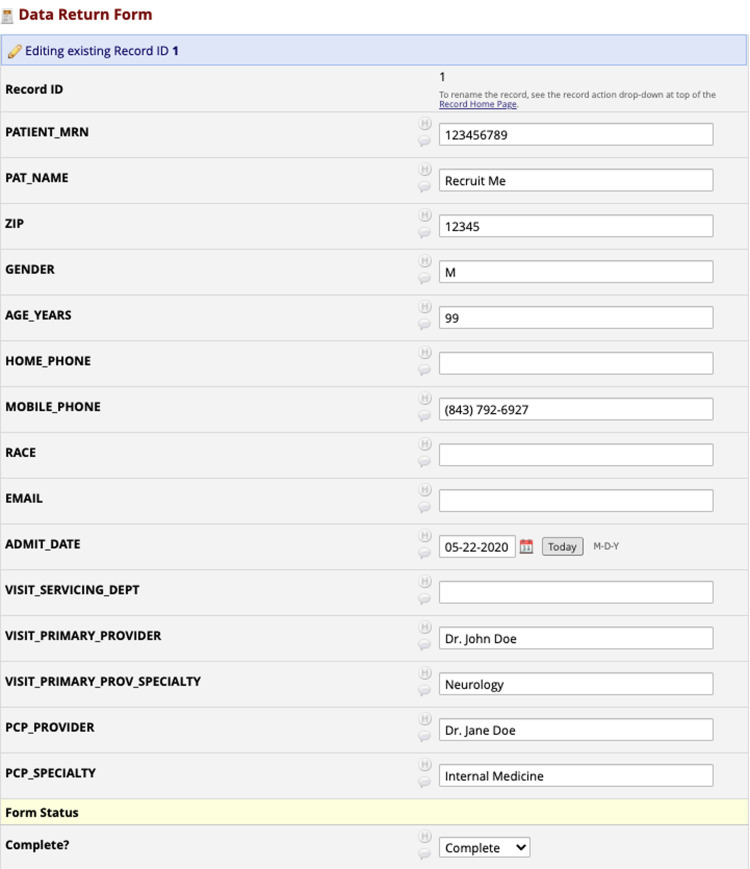
Data return form in REDCap.

**Fig. 3.**
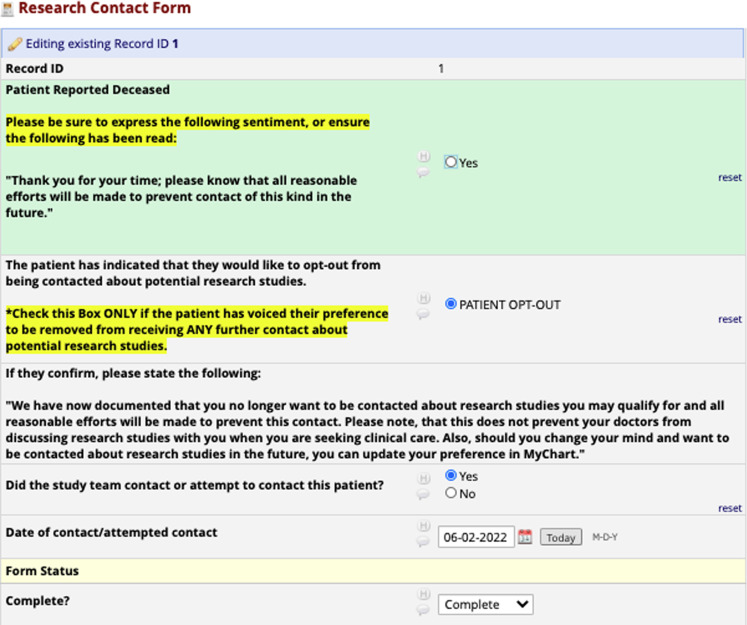
Research contact form in REDCap.

Following IRB approval, a study team may request a recruitment report be developed by BMIC’s honest brokers. These reports are made up of data queried from the institution’s research data warehouse to identify all patients that meet a study’s eligibility criteria who have ***not*** opted-out of being contacted about research. Recruitment reports are accessed by study teams via project-specific REDCap (Research Electronic Data Capture) projects [12,13]. In addition to patient name, Medical Record Number (MRN), and contact information, study teams can request to have particular fields from the medical record displayed for each patient on a unique data return form as shown in Fig. 2. A research contact form, shown in Fig. 3, is affiliated with each patient, on which study teams are required to document date of contact. There are also fields on the form to record if a patient expresses a desire to be removed from all future research contact (not just specific to their study) or to document a notification of patient death. Any notification of patient opt-out preference or death is transferred into the medical record; all active recruitment reports get refreshed to remove these patients and they do not appear on future POR reports. Research teams are allowed to cold contact a patient in any way approved by the IRB, sometimes including automated messaging. Therefore, in some instances, the team will not be informed of a death or a desire to opt-out. Teams are asked to put templated messaging at the bottom of written communication, letting patients know where they can go to learn more about research contact and preferences at MUSC; the teams are also still required to document contact when they reach out to that patient via whichever approved mechanism. All study team members requiring access to a recruitment report must first attend a POR consultation with the research preferences manager to ensure that they are executing the cold contact strategy in line with the institution’s recruitment policy and cold contact SOP and that they are completing the research contact form accurately.

Depending on the specificity of the study criteria or the target enrollment number for a project, there may be significantly more eligible patients identified in a query than are necessary to contact. To limit unnecessary access to patient information, eligible patients are released in batches no greater than 1.5× the study enrollment goal (e.g., a study with an enrollment goal of 200 patients would receive 300 patients delivered to their report at a time). Study teams can request a new batch once they have reviewed and/or contacted everyone from the first group (assuming more patients were identified than appeared on their initial report). Study teams with smaller enrollment goals are the exception, as they are allowed to receive patient reports in batches of 100 (assuming that more potentially eligible patients are identified) to minimize the need for continuous requests for more patients.

There were also initial concerns about over-burdening patients with invitations to participate in research. Based on original PEG feedback, the number of study teams from which a patient could receive contact was limited to three within a 6-month period. A subsequent assessment found that no patient had received their maximum number of contacts nor had any patient complained about being contacted, so this restriction was removed.

### Patient Opt-out Trends

There has been a total of 4804 patients that have chosen to opt-out of research contact since launching POR in January 2021. Patients have primarily opted-out of contact by completing the Research Preferences Questionnaire in their MyChart™ accounts, some prior to having experienced a cold contact recruitment call; only 147 have requested to have a research team opt them out of future contact while being contacted about a particular study. Significantly more females than males opted-out of contact; those who identify as white also opted-out more than those identifying as any other race. These numbers are consistent with the differences in MyChart^™^ users (60.90% of users identify as female and 68.70% of users identify as white) as that was the primary mechanism patients used for opt-out documentation.

### Impact on Study Recruitment

As seen in Table 1, the transition from an opt-in approach to an opt-out approach has resulted in substantial differences in the overall number of potential subjects eligible for contact; researchers can now query from almost the entire patient population. In addition, the demographic data for patients suggests that the pool of potential subjects is more diverse and consistent with the state demographics, increasing the potential for a more representative population for trial recruitment.

**Table 1.** Demographics of patients eligible for contact under opt-in approach and opt-out approach

Total number of patients eligible	Opt-In (*n* = 51,703)	Opt-Out Approach (*n* = 1,564,046)
**Gender, *N* (%)**		
Female	33,670 (65.12)	849,312 (54.30)
Male	18,033 (34.88)	712,593 (45.56)
Unknown/Other*	0(.00)	2,141(.14)
**Race, *N* (%)**		
American Indian or Alaskan Native	108(.21)	2,483 (.16)
Asian	483 (.93)	15,026 (.96)
Black or African American	6,796 (13.14)	432,881 (27.68)
Native Hawaiian or Pacific Islander	39 (.08)	1,357 (0.09)
Other	1,455 (2.81)	191,156 (12.22)
White	42,822 (82.82)	921,143 (58.89)

* Available report for opt-in did not include an “Unknown/Other” category for gender.

During the period from January 2021 to September 1, 2022, recruitment reports were provided to teams for a total of 49 studies. The demographic data of patients appearing on recruitment reports for these studies were also consistent, in some ways, with the overall pool of those eligible for contact; for example, 55.7% of patients appearing on a report were female and 44.2% were male, consistent with gender distribution of those eligible (54.30% female, 45.56% male).

A survey was distributed to study teams using POR to capture the perceived success of study recruitment and diversity of enrolled subjects since implementation of the cold contact strategy; surveys were completed for 21 studies. Teams were asked to identify the perceived success of recruitment on a sliding scale from “not successful” to “somewhat successful” to “very successful.” For 16 of the 21 projects, study teams identified their recruitment as falling somewhere between “somewhat successful” and “very successful.” In the survey’s comment section, one team wrote this of their experience recruiting for a study using POR as their only strategy: “Phase I consisted of *n* = 50. We met our enrollment goal within one month and completed Phase I within 3 months.” To capture perceived success at meeting subject diversity goals, teams again used a sliding scale with the endpoints being “not meeting diversity goals” and “exceeding diversity goals,” and the center of the scale representing “meeting diversity goals.” For 12 of the 21 projects, study teams identified their project as falling somewhere between “meeting diversity goals” and “exceeding diversity goals.” One researcher commented, “I was afraid that I was not going to reach a diverse group of people but I was very wrong and my most diverse recruits came from POR methods.”

## Discussion

The implementation of an opt-out, cold contact approach to research recruitment clearly achieves the goals of offering a larger, more diverse, and likely more representative population for potential participation in research than previous strategies. MUSC’s change in process from an opt-in to an opt-out approach yielded >3000% increase in the number of patients within the potential recruitment pool. In addition, the overall pool of potential study subjects better mirrored the overall population of South Carolina.

Although many institutions have expressed discomfort with an opt-out policy under the premise that it conflicts with patient privacy or adds undue burden to patients, this approach actually satisfies important ethical concerns regarding research recruitment. These ethical issues have been described previously and include autonomy, beneficence, and justice [3]. Recruitment policies that more stringently restrict cold contact may actually limit patient choice. For example, limiting access to patients through their physicians may result in gatekeeping bias (providers preventing patient access to research) that does not align with true patient preferences [3]. It was discovered through the community PEGs that patients wanted to know about research opportunities. Access to a limited pool of potential subjects may risk under-enrollment or selection bias and may also reduce the generalizability of research findings and create an excess burden on the participants who opted-in for research. These aforementioned scenarios pose risks to both the beneficence and justice components of ethical research.

Patients assuring their control over research involvement is critical to the concept of patient autonomy. This is managed by providing the opportunity to opt-out for both the study they were contacted for and, also, future research contacts. Patients are given multiple methods to convey their preference, which is easily trackable and implemented when generating recruitment reports. Only a minority of participants contacted for a research study elected to opt-out of future contact. Patients also have the ability to reverse that decision to once again be eligible for contact about research opportunities. This process, notably, does not restrict clinician investigators from discussing research opportunities with their own patients. In addition to assuring their control over research involvement, the concept of autonomy in research ethics also holds the assumption that patients will be presented with enough information to make informed choices and decisions. Prior to implementing opt-out, many patients were not provided with information regarding potential research study opportunities nor given the option to participate should they desire.

While the researcher survey responses offered some anecdotal evidence that MUSC’s implementation of the POR strategy and the opt-out approach to documenting research contact preference has improved study recruitment as well as aided in the diversification of the participant populations, there has not been a way of confirming this association through enrollment data. On March 1, 2022, MUSC began requiring study teams that met certain criteria to enter study information, including subject accrual data, into its instance of OnCore^™^, MUSC’s enterprise research clinical trial management system (CTMS). Studies that are recruiting using the POR strategy meet the criteria for mandatory documentation in OnCore^™^. It is expected that, with time, enough data will be entered for both studies utilizing POR and not, that inferences can be made about the impact of this strategy on successful enrollment and diversity.

The researcher survey responses, though limited, suggest that some study teams using POR perceived their recruitment as lacking in success (whether in number of patients, diversity of sample, or both). It should be noted, however, that studies for which responses were collected varied in the degree to which POR was utilized over other recruitment strategies. The type of study and method used for initial contact, though not captured in the survey responses, likely also played a factor in the success of POR implementation. Study teams are currently using varying cold contact methods (phone call, email, letter, MyChart^™^, etc.). Some may use multiple methods or make more than one contact attempt. This variation may contribute to differences in perceived success of the POR process.

### Next Steps

Data collected via the CTMS will be used to track how the POR strategy impacts study enrollment and sample diversity (particularly as compared studies not using POR); trends will also be identified to determine if study types and methods of contact impact the success of POR as a strategy with various patient groups. This information can be used to enhance POR report release consultations to offer more study-specific guidance and strategy, ensuring the approach is being used most efficiently and successfully. Specifically, should it be discovered that, despite increased access to a more representative patient population, the patients contacted and ultimately enrolled in studies using POR are still lacking in diversity, the POR team will give researchers feedback and tools to overcome that limitation (i.e., filtering reports by various demographics so efforts can be better target at those harder-to-reach groups).

While the enrollment data will be enlightening, capturing continuous feedback from the community and patient population, as was done at the start of the process, is critically important to fine-tuning implementation of the cold contact strategy and staying true to the preservation of patient autonomy. For these reasons, PEGs will be held continuously to capture and understand community opinion and perception, as well as to gain feedback on messaging for the POR process both generally and at the protocol level.

## Supplementary material

For supplementary material accompanying this paper visit https://doi.org/10.1017/cts.2023.21. click here to view supplementary material
