# Supplementary material for: Enhancing study recruitment through implementation of an opt-out, cold contact process with consideration for autonomy, beneficence and justice
Source: J Clin Transl Sci. 2023 Feb 8;7(1):e63. doi: 10.1017/cts.2023.21 (PMC10052414; doi:10.1017/cts.2023.21)
Supplement: Supplementary file 1 [file S2059866123000213sup001.pdf]

University Policy

Section # ** N/A	Policy # U-IRB-HRPP 7.8	IRB HRPP 7.8 Recruitment of Research Participants	
**Responsible Department: Office of Research Integrity**			
**Date Originated** Not Set	**Last Reviewed** Not Set	**Last Revised** **01/21/2021**	**Effective Date *** 10/07/2021

Printed copies are for reference only. Please refer to the electronic copy for the official version.

I. Introduction

The policy applies to all research recruitment activities by MUSC investigators. The Institutional Review Board (IRB) will evaluate the plan for recruitment of research participants in accordance with all relevant laws and regulations.

Recruitment is considered the start of the participant selection process and is a prelude to the informed consent/assent process. Investigators must respect an individual's reasonable expectation for privacy when considering how information is gathered about a potential participant and who will invite the individual to participate in the research. Investigators must ensure that recruitment activities are free of bias, do not exert undue influence on or coerce a potential participant to volunteer, or imply a guarantee of benefits beyond what is outlined in the protocol and consent form approved by the IRB.

II. Policy

Recruitment methods used to solicit volunteers into human research must be equitable and free of bias, undue influence and coercion and must respect the privacy of potential research participants. The IRB must review and approve the methods, materials, procedures, and tools used to recruit potential research participants before they are implemented.

A. Screening, Recruiting, or Determining Eligibility (45 CFR 46.116(g)

An IRB may approve a research proposal in which an investigator will obtain information or biospecimens for the purpose of screening, recruiting, or determining the eligibility of prospective subjects without the informed consent of the prospective subject or the subject's legally authorized representative, if either of the following conditions are met:

1) The investigator will obtain information through oral or written communication with the prospective subject or legally authorized representative, or

2) The investigator will obtain identifiable private information or identifiable biospecimens by accessing records or stored identifiable biospecimens.

B. Contacting Prospective Participants Who Were Identified from Medical Records

* Policies become effective on the date of publication
** If not applicable, enter NA

MUSC is an "opt-out" institution, meaning that MUSC patients may be contacted about research opportunities unless they have chosen to "opt-out" of this potential contact by indicating their preference in their medical record.

The IRB will approve recruitment plans involving "cold calling" participants only where this method of contact is deemed appropriate in regard to the nature of the study. "Cold calling" is when an individual is contacted by someone they do not know and the contact is unexpected.

i. ***When "Cold Calling" <u>IS NOT</u> Approved for a Study***

If the IRB has not approved a study for "cold-calling" PIs or research staff who are not directly involved in a patient's clinical care, may NOT use this method of recruitment.

The research team may request that a subject's treating clinician present his/her patient with information about the opportunity to participate in research. The clinician may directly (e.g. during an office visit) or indirectly (e.g. by IRB-approved letter, email, flyer) provide information about the research study, and/or provide a contact number for the PI or research staff to the patient. Study information provided by a clinician should not include any language or information that may be perceived as unduly influencing or coercive, or imply that medical care could be influenced by choice to participate or not. If the patient is interested in the research study, then he/she contacts the research team for more information. Patient information may be released to the PI or research staff only if a potential participant gives permission for his/her identifying information to be shared.

ii. ***When "Cold Calling" <u>IS</u> Approved***

If the IRB has approved a study for "cold calling" PIs or research staff may contact any patients who have ***not*** documented an "opt-out" of research contact preference. Patients' documentation of their "opt-out" preference will be noted in their electronic health record. Specified methods of contact will be approved by the IRB, and documentation of every patient contacted must be recorded by the study team unless an exception is granted by the IRB.

The IRB may approve cold-calling methods of recruitment if:

- The researchers provide justification in the IRB application as to why this strategy is appropriate
- The investigator must provide a recruitment script for IRB review that will be used when cold calling patients for potential research participation.

C. Secondary Recruitment

Secondary recruitment methods are used when investigators wish to recruit specific groups of participants through friends and family of existing participants. Some forms of this type recruitment are often called "snowball recruiting". To protect the privacy of those

* Policies become effective on the date of publication
** If not applicable, enter NA

being recruited, the IRB will consider whether these methods are appropriate for the study.

Participant-initiated Secondary Recruitment is when the investigator asks current subjects to pass along recruitment materials or information to friends and family. The participant initiates the contact with family and friends, and these people must then contact the research team if they wish to learn more about the study. If a referred individual contacts the research team and is interested and eligible (regardless of whether or not they agree to participate), the referring participant may receive a small amount of payment for the referral (see below). "Snowball recruiting" may be done in a number of ways. The details of this process must be articulated in the investigator's IRB application. The IRB will review the process, which will be approved on a case-by-case basis, depending on the nature of the study.

Payment for Secondary Recruitment. It is permissible to allow this form of recruitment for studies by providing cash, gift certificates or other incentives to subjects to promote their aid in recruiting new individual subjects into studies. The incentive to the subject (as a recruiter) must be defined with moderation, justice and autonomy. The incentive should be based on local norms and must be approved by the IRB.

D. Future Recontact

During the consent process, investigators may ask potential participants if they would be willing to be recontacted for future research studies. The response to this should be documented in the informed consent. It is then permissible for the investigator to include the name and contact information of that individual for future studies. This identifiable information should not be shared outside the research team. This list of individuals who are willing to be contacted for research purposes should not be stored with health information. Future research contact decisions documented in specific study consent forms, are separate and distinct from their opt-out research contact preference included in their medical record.

E. Research Involving MUSC Employees or MUSC Students

For specific guidance see HRPP 8.6 Research Involving MUSC Employees and Students

F. Recruitment Materials

For specific guidance see HRPP 7.2 - Advertisements for Research Participants Policy and Procedures

G. Finders Fees

Finder's fees are defined as payments to physicians, nurses, or other health-care professionals for the mere recruitment of research participants. The MUSC IRB does not allow the use of finders' fees in research. For specific guidance see HRPP 7.4 – Recruitment Incentives Policy and P
